# Diversification of the insulin-like growth factor 1 gene in mammals

**DOI:** 10.1371/journal.pone.0189642

**Published:** 2017-12-14

**Authors:** Peter Rotwein

**Affiliations:** Department of Biomedical Sciences, Paul L. Foster School of Medicine, Texas Tech University Health Sciences Center, El Paso, Texas, United States of America; Universitat de Barcelona, SPAIN

## Abstract

Insulin-like growth factor 1 (IGF1), a small, secreted peptide growth factor, is involved in a variety of physiological and patho-physiological processes, including somatic growth, tissue repair, and metabolism of carbohydrates, proteins, and lipids. *IGF1* gene expression appears to be controlled by several different signaling cascades in the few species in which it has been evaluated, with growth hormone playing a major role by activating a pathway involving the Stat5b transcription factor. Here, genes encoding *IGF1* have been evaluated in 25 different mammalian species representing 15 different orders and ranging over ~180 million years of evolutionary diversification. Parts of the *IGF1* gene have been fairly well conserved. Like rat *Igf1* and human *IGF1*, 21 of 23 other genes are composed of 6 exons and 5 introns, and all 23 also contain recognizable tandem promoters, each with a unique leader exon. Exon and intron lengths are similar in most species, and DNA sequence conservation is moderately high in orthologous exons and proximal promoter regions. In contrast, putative growth hormone-activated Stat5b-binding enhancers found in analogous locations in rodent *Igf1* and in human *IGF1* loci, have undergone substantial variation in other mammals, and a processed retro-transposed *IGF1* pseudogene is found in the sloth locus, but not in other mammalian genomes. Taken together, the fairly high level of organizational and nucleotide sequence similarity in the *IGF1* gene among these 25 species supports the contention that some common regulatory pathways had existed prior to the beginning of mammalian speciation.

## Introduction

Insulin-like growth factor 1 (IGF1) is a 70-residue, secreted protein that along with IGF2 and insulin comprises a conserved protein family found in most mammalian species and in many other vertebrates [1–4]. IGF1 plays a central role in pre- and post-natal growth in human children and in juveniles of other mammals as a key mediator of the actions of growth hormone (GH) [5–9], and also is involved in control of intermediary metabolism, in tissue repair, and in disease pathogenesis throughout life [10–13].

**Fig 1:**
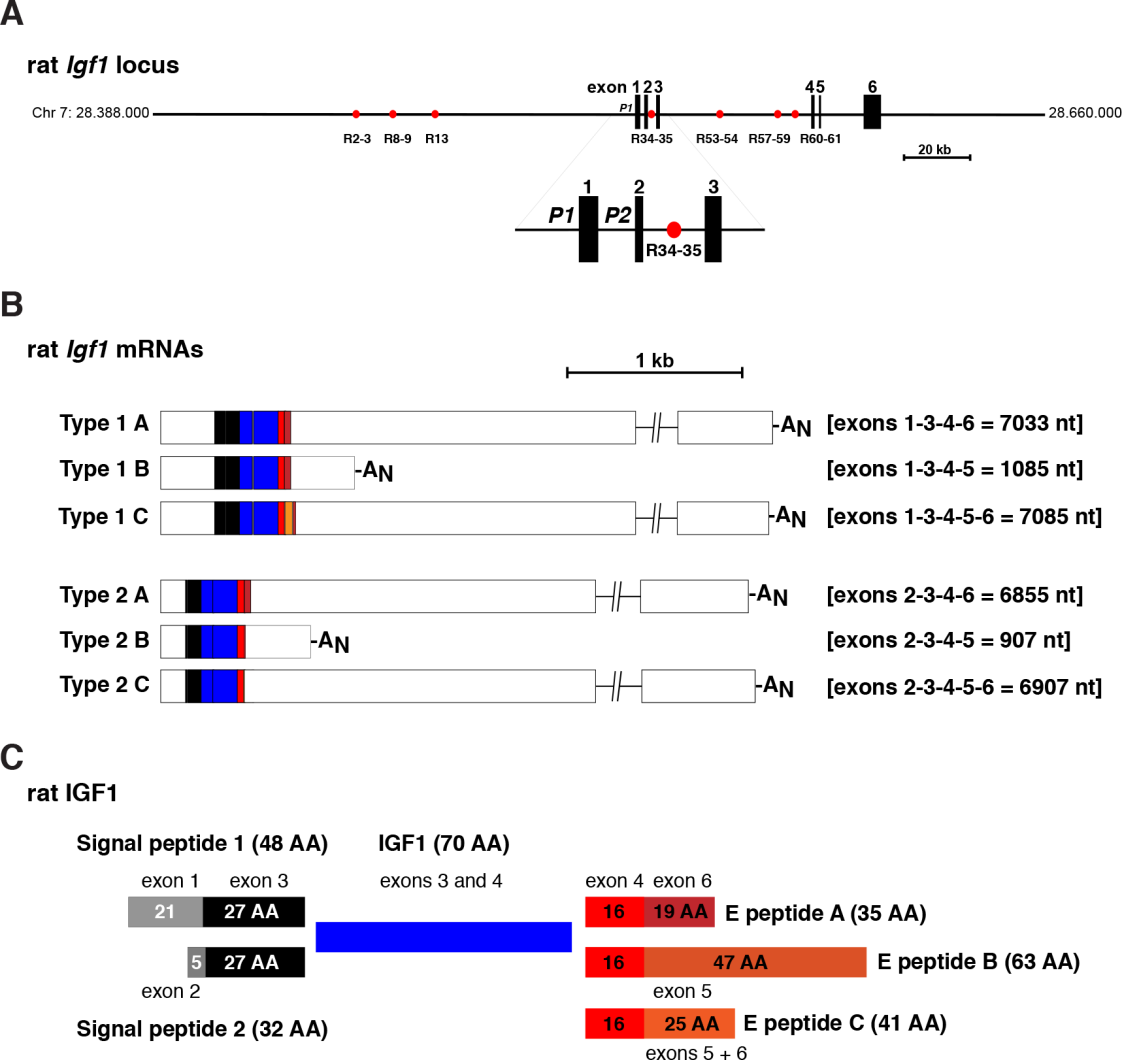
Organization of the rat *Igf1* gene and mRNAs. **A**. Map of the rat *Igf1* locus with chromosomal coordinates. Exons are depicted as boxes, and introns and flanking DNA as horizontal lines. A scale bar is shown. The enlargement below the main map illustrates the two *Igf1* promoters, P1 and P2, and exons 1–3. The red circles depict previously identified locations for GH-inducible binding sites for the Stat5b transcription factor. **B**. Diagram of the six major classes of rat *Igf1* mRNAs. Type 1 contains exon 1 (transcribed by *Igf 1* promoter 1), and Type 2 encodes exon 2 (transcribed by promoter 2). The letters A, B and C indicate transcripts that contain alternatively processed exon 6, exon 5, or exons 5 and 6, respectively. The average length of each mRNA class is indicated in nucleotides (nt). A_N_ represents the polyadenylic acid tail at the 3’ end of mRNAs. **C**. Diagram of rat IGF1 protein precursors, showing the derivation of each segment from different *Igf1* exons. Mature, 70-amino acid IGF1 (center) is found within all precursors; signal peptides 1 and 2 (left), and E peptides A, B and C (right), are alternatively encoded in part by different *Igf1* mRNAs.

Limited analyses suggest that *IGF1* genes are conserved among mammals [1, 2, 14]. Two gene promoters have been shown to control *IGF1* gene expression in the few mammals in which it has been studied experimentally, [2, 15–20]. In these species, *IGF1* genes are composed of six exons and five introns and are of similar size [2, 20]. For example, the human *IGF1* gene spans ~85.1 kb on chromosome 12q23.2, and the single-copy rat and mouse *Igf1* genes are ~79.3 and ~78.0 kb in length, respectively [21]. *Igf1* gene expression has been studied most extensively in rats, where it has been demonstrated that multiple *Igf1* mRNAs are produced by the combination of gene transcription from two promoters, initiation of mRNA synthesis at several sites in each promoter-specific leader exon, differential RNA splicing involving exons 5 and 6, and alternative polyadenylation at the 3’ end of exon 6 [15, 16, 19, 22, 23] (Fig 1A and 1B). It is presumed that similar events occur in other mammals, although experimental data are limited [24, 25]. Six classes of IGF1 protein precursors potentially result from the translation of the many rat *Igf1* mRNAs [2] (Fig 1C). Although these molecules differ in the NH_2_-portions of their signal peptides, and in the COOH-terminal parts of their extension peptides or E domains, they all encode the identical 70-amino acid, biologically active, and secreted mature IGF1 protein [2, 26].

Consistent with its physiological role in somatic growth, GH is a critical regulator of *IGF1* gene expression in mammals [5, 18, 27–31]. GH, acting through its trans-membrane receptor and the intracellular tyrosine protein kinase, Jak2 [32–35], acutely activates the Stat5b transcription factor [36], which then binds to multiple transcriptional enhancers that are found in chromatin throughout the *Igf1* locus, leading to stimulation of the two *Igf1* promoters in rat liver [37, 38], and presumably in other organs and tissues. In humans, a similar pathway has been identified in limited studies in cultured cells, although to date fewer GH-inducible and Stat5b-binding putative enhancers have been functionally detected in the human *IGF1* locus than in the rat [39]. Since inactivating mutations in the human *STAT5B* gene have been characterized that are associated with growth failure and IGF1 deficiency [40–42] and genetic loss of *Stat5b* leads to growth deficiency in mice [43, 44], it seems likely that this molecular pathway has been conserved between rodents and humans.

The present studies were initiated in order to understand the breadth and depth of both conservation and variation in *IGF1*/*Igf1* genes in mammals as a means of gaining insight into key aspects of gene regulation as it has evolved during speciation. Using the information found within publically available databases, *IGF1* loci and genes have been analyzed in 25 mammalian species representing 15 orders and spanning ~180 million years (Myr) of evolutionary diversification. The results demonstrate substantial conservation in coding regions of exons and in overall exon, intron, and proximal gene promoter topology, leading to the idea that common paradigms governing *IGF1* gene regulation were present at the onset of the mammalian radiation.

## Materials and methods

### Genome database searches

Mammalian genomic databases were accessed using the Ensembl Genome Browser (www.ensemble.org) [21]. Searches were conducted using BlastN or BlastP under normal sensitivity, with rat *Igf1* gene DNA segments (*Rattus norvegicus*, genome assembly Rnor_6.0), or protein sequences (from the National Center for Biotechnology Information Protein database) as initial queries, respectively. Additional searches were performed in Ensembl with other mammalian species DNA sequences as queries to follow-up and verify initial results. In addition, when relevant information could not be found in the Ensembl Genome Browser, both genomic DNA and gene expression data files were searched using the Sequence Read Archive of the National Center for Biotechnology Information (SRA NCBI; www.ncbi.nlm.nih.gov/sra/), and for human *IGF1*, RNA data from the Portal for the Genotype-Expression Project (GTEx V7, https://www.gtexportal.org/home/). The following genome assemblies were examined: armadillo (*Dasypus novemcinctus*, Dasnov3.0), cat (*Felis catus*, Felis_catus_6.2), chimpanzee (*Pan troglodytes*, CHIMP2.1.4), cow (*Bos taurus*, UMD3.1), dog (*Canis lupus familiaris*, CanFam3.1), dolphin (*Tursiops truncates*, turTru1), elephant (*Loxodonta Africana*, loxAfr3), gibbon (*Nomascus leucogenys*, Nleu1.0), guinea pig (*Cavia porcellus*, cavPor3), human (*Homo sapiens*, GRCh38), megabat (*Pteropus vampyrus*, pteVam1), microbat (*Myotis lucifugus*, Myoluc2.0), mouse (*Mus musculus*, GRCm38), opossum (*Monodelphis domestica*, BROADO5), orangutan (*Pongo abelii*, PPYG2), pig (*Sus scrofa*, Sscrofa10.2), platypus (*Ornithorhynchus anatinus*, OANA5), rabbit (*Oryctolagus cuniculus*, OryCun2.0), sheep (*Ovis aries*, Oar_v3.1), sloth (*Choloepus hoffmanni*, choHof1), squirrel (*Ictidomys tridecemlineatus*, spetri2), Tasmanian devil (*Sarcophilus harrisii*, DEVIL7.0), tree shrew (*Tupaia belangeri*, TREESHREW), and wallaby (*Macropus eugenii*, Meug_1.0). GENCODE/Ensemble databases were searched for protein sequences. In all outcomes, the highest scoring results mapped to the respective *IGF1/Igf1* gene, locus, or protein. In text and Tables, results are reported as percent identity over the entire query region, unless otherwise specified.

## Results

### Nomenclature and experimental strategy

Naming conventions adopted here include the term ‘*Igf1*’ for rodent genes and mRNAs, ‘*IGF1*’ for human, other primate, and all other mammalian genes and transcripts, and ‘IGF1’ for all proteins. As a preliminary examination of mammalian *IGF1*/*Igf1* genes and mRNAs within Ensembl revealed that most assignments were incomplete when compared with rat *Igf1* or human *IGF1*, thus limiting the value of using the data for comparative analyses, a major experimental goal was to map all genes as thoroughly as possible. An iterative strategy was developed, in which homology searches first were conducted with segments of the rat *Igf1* gene, followed by secondary searches using either components of human *IGF1* or other genes that were evolutionarily more similar to specific target species, with a final follow-up using the resources of the SRA NCBI to identify *IGF1* gene segments not detected in Ensembl. As revealed below, results revealed substantially higher levels of gene complexity than described in the data curated by Ensembl.

### *IGF1* genes in mammals

*IGF1* appears to be a 6-exon, 5-intron gene in 23 of 25 mammalian genomes studied here (Table 1), and the presumptive overall structure resembles that of rat *Igf1* or human *IGF1* in the vast majority (Table 1). Exceptions include guinea pig, in which an exon 5 was not identified, and wallaby, in which no exon 3 was identified, and in which intron 5 was not found (the latter differences may reflect both poor quality DNA sequence data and the fact that in the wallaby genome the *IGF1* locus has not been mapped yet to a single continuous DNA segment) (Table 1). There was reasonable congruence in the lengths of different gene components among the 23 species recognized to have 6 exons and 5 introns (Table 1), and total gene sizes ranged from 71,136 bp in the microbat to 119,762 bp in the opossum (Table 1). For 20 of these genes their lengths were within ±10% of rat *Igf1* at 79,281 bp. Nearly all of the variation in the outliers (megabat, microbat, opossum, Tasmanian devil) could be attributed to introns 5 and/or 3, the largest *IGF1* introns, which were measured at ~60% and ~33% longer respectively than the mean in opossum and Tasmanian devil, and were ~20% shorter in megabat and microbat (Table 1).

**Table 1:** Organization of mammalian IGF1 genes*.

Species	Exon 1	Intron 1	Exon 2	Intron 2	Exon 3	Intron 3	Exon 4	Intron 4	Exon 5	Intron 5	Exon 6	Length
Rat	343	1691	165	3179	157	50629	182	1402	≥52	15130	6351	79281
Mouse	343	1810	164	3345	157	48731	182	1525	≥52	15325	6386	78007
Rabbit	343	1654	163	3280	157	48887	182	1526	≥162	≤14231	6358	76943
Squirrel	343	1730	165	3350	157	47382	182	1483	≥117	≤13503	6191	74538
Guinea pig	332	1539	161	3238	157	44620	182	-	nd	16408	6188	72949
Cow	343	1658	163	2651	157	51296	182	1429	392	13368	6455	78091
Dolphin	343	1643	160	2297	157	50565	182	1443	≥189	≤13282	6350	76713
Pig	343	1659	163	2717	157	50972	182	1693	354	14635	6351	79226
Sheep	343	1652	163	2658	157	52894	182	1471	≥116	≤13935	6356	79927
Tree shrew	343	1670	163	2559	157	54955	182	-	52	19668	6144	85899
Human	327	1688	165	2688	157	55953	182	1506	513	14925	6992	85096
Chimpanzee	327	1671	165	2681	157	54975	182	1506	514	14888	6988	84054
Gibbon	343	1664	163	2651	157	55232	182	1474	≥189	≤15165	6696	83947
Orangutan	343	1668	163	2721	157	54843	182	1506	≥189	≤15241	6712	83678
Cat	343	1767	165	2714	157	50029	182	1506	≥162	≤13747	6365	77100
Dog	343	1716	164	2690	157	51456	182	1466	≥163	≤14060	6565	79001
Elephant	344	1673	160	3223	157	55621	182	1606	≥231	≤15293	6612	85101
Sloth	343	1636	163	3131	157	50154	182	1598	≥186	≤13195	6305	77277
Megabat	343	-	153	3451	157	49105	182	1478	≥186	≤11992	4912	71837
Microbat	343	1620	162	2519	157	46725	182	1367	≥162	≤11800	6102	71136
Armadillo	343	1606	162	2622	157	51288	182	1342	≥222	≤12232	6162	76429
Opossum	343	1869	164	3948	157	80162	182	2495	≥244	≤23758	6406	119762
Platypus	342	1979	107	2091	157	59105	182	1089	≥252	≤17697	≥59	82661
Tas. devil	344	2143	160	3614	157	78727	182	2866	≥234	≤20068	6513	115074
Wallaby	344	1757	162	-	nd	19807	182	2832	≥170	nd	≥2301	-

*length is in base pairs

nd—not detected

dash indicates that information is not available

DNA conservation was generally extensive for exons 1–4 among the species studied, with overall nucleotide sequence identities with different rat exons ranging from a high of 94–98% for mouse, to a low of 82–92% for platypus (Table 2). The lengths of these exons also were conserved among the genomes analyzed (Table 1). In contrast, exons 5 and 6 were more variable, with exon 5 being of different lengths because of its involvement in alternative splicing in most of the 25 species analyzed, as illustrated for rat *Igf1* (Fig 1B). The total length of exon 6 was comparable in 22 of 25 species based on mapping of locations of DNA homology with rat *Igf1* or human *IGF1* (Table 1; exceptions are megabat, platypus, wallaby), although the overall extent of nucleotide sequence identity with the corresponding rat exon was fairly limited (Table 2). DNA similarity was nearly as high for both *Igf1* promoters as for exons 1–4, ranging from 85–92% for promoter 1 and 75–98% for promoter 2, although as observed for exons 5 and 6, the length of sequence homology was variable among different species (Table 3), perhaps indicating that diversification of proximal promoter regulatory elements has occurred during mammalian speciation (but see below).

**Table 2:** Nucleotide identity with rat *Igf1* exons (%).

Species	Exon 1 (343 bp)	Exon 2 (165 bp)	Exon 3 (157 bp)	Exon 4 (182 bp)	Exon 5 (52 bp)	Exon 6 (6351 bp)
Mouse	98	94	95	94	92 (52 bp)	88 (6197 bp)
Rabbit	94	87	89	90	80 (162 bp)	86 (1616 bp)
Squirrel	95	88	91	88	78 (≥117 bp)	85 (1637 bp)
Guinea pig	86	89	86	87	nd	92 (613 bp)
Cow	94	90	86	86	82 (392 bp)	85 (1214 bp)
Dolphin	94	88	84	87	82 (189 bp)	86 (1389 bp)
Pig	94	87	87	90	59 (354 bp)	87 (1367 bp)
Sheep	94	90	85	86	45 (116 bp)	88 (1155 bp)
Tree shrew	95	90	89	89	75 (52 bp)	86 (1535 bp)
Human	94	90	87	88	80 (513 bp)	84 (2097 bp)
Chimpanzee	94	90	87	88	80 (514 bp)	85 (2062 bp)
Gibbon	94	91	88	89	80 (189 bp)	85 (1768 bp)
Orangutan	94	90	87	88	80 (189 bp)	83 (1960 bp)
Cat	94	90	88	88	78 (162 bp)	88 (1546 bp)
Dog	95	90	90	89	76 (163 bp)	88 (1129 bp)
Elephant	94	87	87	90	60 (231 bp)	88 (1388 bp)
Sloth	94	89	87	87	76 (186 bp)	86 (1409 bp)
Megabat	95	86	88	88	72 (186 bp)	86 (1058 bp)
Microbat	95	89	90	88	80 (162 bp)	86 (909 bp)
Armadillo	94	87	90	88	78 (222 bp)	86 (1339 bp)
Opossum	89	88	88	84	76 (244 bp)	85 (311 bp)
Platypus	92	85	82	82	65 (252 bp)	92 (59 bp)
Tas. devil	89	87	85	93	76 (234 bp)	87 (425 bp)
Wallaby	88	86	ndx	84	49 (170 bp)	87 (274 bp)

nd–not detected; ndx–not detected because of poor quality DNA sequence

**Table 3:** Nucleotide identity with rat *Igf1* promoters (%).

Species	Promoter 1 (540 bp)	Promoter 2 (592 bp)
Mouse	87 (540 bp)	88 (592 bp)
Rabbit	89 (133 bp)	90 (121 bp)
Squirrel	87 (242 bp)	98 (96 bp)
Guinea pig	90 (144 bp)	86 (97 bp)
Cow	87 (156 bp)	96 (120 bp)
Dolphin	88 (144 bp)	96 (113 bp)
Pig	89 (156 bp)	88 (188 bp)
Sheep	90 (119 bp)	96 (120 bp)
Tree shrew	88 (193 bp)	93 (137 bp)
Human	87 (242 bp)	88 (195 bp)
Chimpanzee	88 (231 bp)	88 (195 bp)
Gibbon	88 (242 bp)	87 (224 bp)
Orangutan	88 (231 bp)	92 (149 bp)
Armadillo	86 (195 bp)	93 (121 bp)
Cat	91 (193 bp)	95 (122 bp)
Dog	86 (242 bp)	93 (121 bp)
Elephant	89 (168 bp)	94 (121 bp)
Sloth	89 (193 bp)	94 (121 bp)
Megabat	87 (244 bp)	90 (219 bp)
Microbat	87 (194 bp)	95 (121 bp)
Armadillo	86 (195 bp)	93 (121 bp)
Opossum	85 (151 bp)	91 (109 bp)
Platypus	89 (138 bp)	75 (217 bp)
Tasmanian devil	91 (148 bp)	90 (115 bp)
Wallaby	92 (83 bp)*	90 (108 bp)

*poor quality DNA sequence

### Conservation in IGF1 protein sequences among mammals

**Fig 2:**
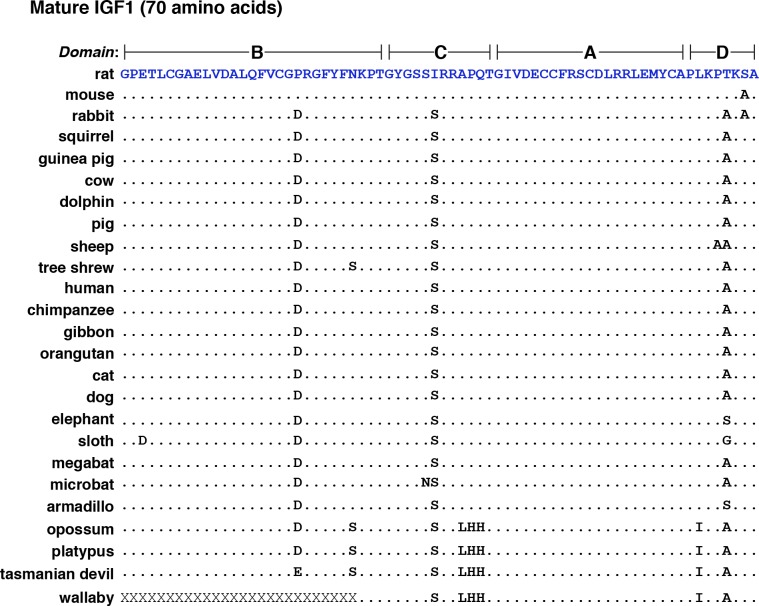
Alignments of mammalian IGF1 proteins. Amino acid sequence of IGF1 from 25 mammalian species in single letter code. Identities are indicated by dots, and differences among species are depicted. X depicts an unknown amino acid. The color of rat IGF1 amino acids corresponds with the map in Fig 1C.

The 70-residue secreted IGF1 molecule is encoded within six different types of protein precursors (Fig 1C) that differ at their NH_2_- and COOH-termini through a combination of mechanisms that produce many classes of *Igf1* mRNAs in the rat (Fig 1B). Mature IGF1 in turn is divided into four domains, termed B, C, A, and D, with the first three being analogous to the B and A chains of insulin, and the C-peptide of pro-insulin (Fig 2) [45]. Among the mammals studied, 70-amino acid IGF1 was not identical to the rat protein in any species (Table 4), although a single conservative substitution was seen in the mouse (Ser^69^ to Ala, Fig 2). In all other mammals, at least three differences with rat IGF1 were found, with the most prevalent alterations being Pro^20^ to Asp, Ile^35^ to Ser, and Thr^67^ to Ala (Fig 2). IGF1 was identical in 12 species: squirrel, guinea pig, cow, dolphin, pig, human, chimpanzee, gibbon, orangutan, cat, dog, and megabat; moreover rabbit, sheep, tree shrew, microbat, and armadillo IGF1 each varied by a single residue from this group (Fig 2). The only other mammals with more divergent IGF1 molecules were the three marsupials (opossum, Tasmanian devil, wallaby) and the one monotreme (platypus), whose genome sequences predicted mature IGF1 proteins with eight differences from rat IGF1, and five from other species, although wallaby IGF1 was incomplete (Fig 2).

**Table 4:** Amino acid identities with rat IGF1 (%).

Species	Signal peptide N1 (21 AA)	Signal peptide N2 (5 AA)	Signal peptide C (27 AA)	Mature IGF-I (70 AA)	Common E (16 AA)	E_A_ peptide (19 AA)	E_B_ peptide (47 AA)	E_C_ peptide (25 AA)
Mouse	100	60	96	99	100	100	74	80
Rabbit	90	none	85	94	94	95	57*	none
Squirrel	90	none	89	96	94	90	51*	88
Guinea pig	90	100	70	96	94	90	none	none
Cow	90	40	81#	96	88	95	53*	76
Dolphin	90	40	81	96	81	90	55*	76
Pig	90	40	85	96	88	90	53*	76
Sheep	90	40	74	94	88	95	49*	40*
Tree shrew	90	40	89	94	94	84	none	68
Human	90	40	89	96	94	90	53*	64*
Chimpanzee	90	40	89	96	94	90	55*	68
Gibbon	90	40	89	96	94	90	55*	68
Orangutan	90	40	89	96	94	95	55*	72
Cat	90	40	85	96	88	90	45*	36
Dog	90	40	78	96	88	95	43*	64
Elephant	90	40	89	96	88	47	17*	72*
Sloth	90	40	77^	94	88	90	57*	72
Megabat	90	none	93	96	88	90	49*	36*
Microbat	86	40	89	94	81	90	53*	68
Armadillo	90	40	93	96	88	90	60*	72
Opossum	67	none	78	89	88	68	49*	56*
Platypus	76	none	74	89	88	84	17*	52*
Tas. devil	67	none	81	89	88	63	45*	60*
Wallaby	67	none	undefined	86¶	88	none	23*	none

*E_B_ or E_C_ domains are different length than rat (see Figs 4 and 5).

#28 AA

^26 AA

¶only 40 AA identified

**Fig 3:**
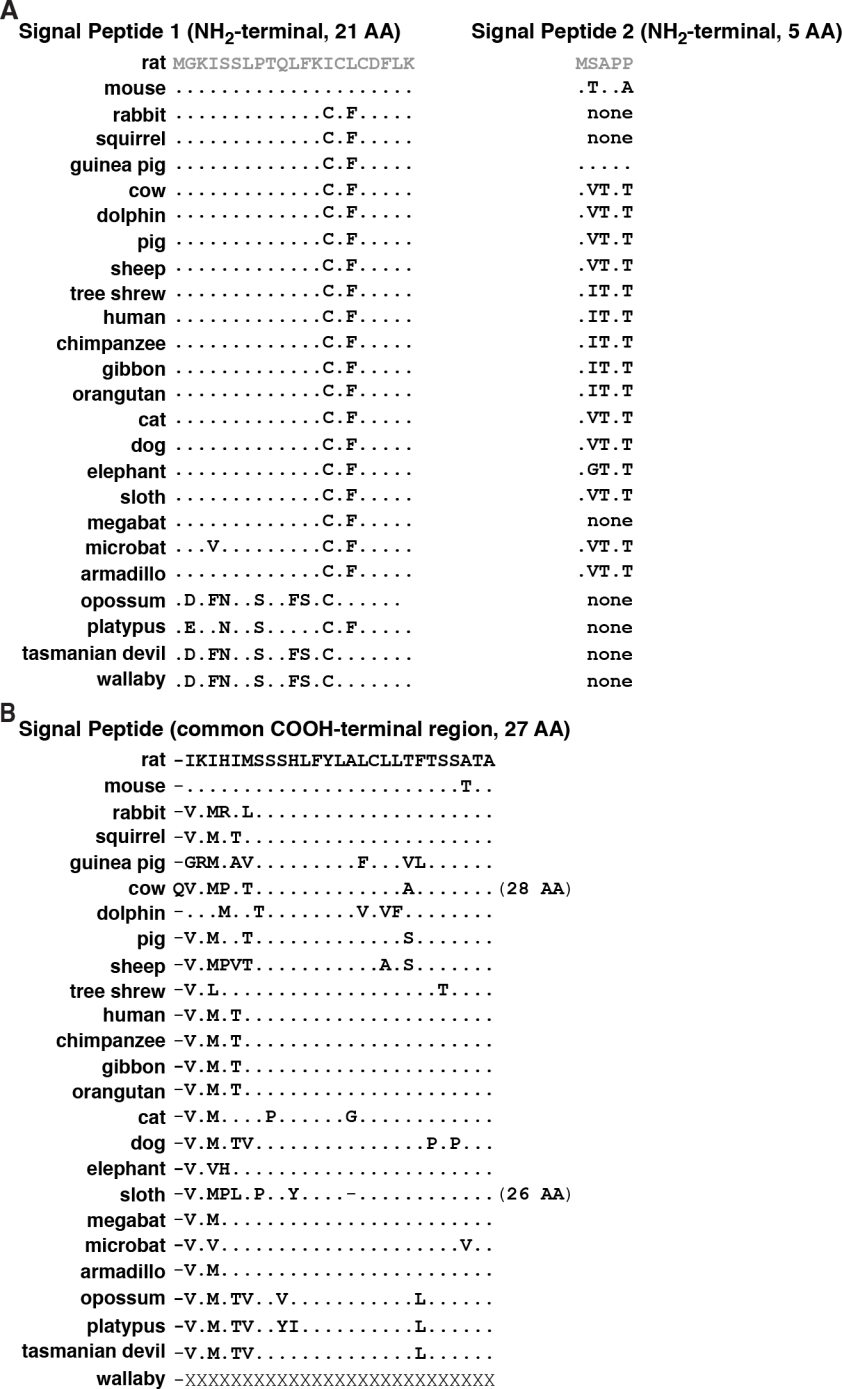
Alignments of mammalian IGF1 signal peptides. Amino acid sequences of IGF1 signal peptides. **A**. The initial 21 residues of signal peptide 1 are encoded by *IGF1*/*Igf1* exon 1. **B**. The first 5 residues of signal peptide 2 are derived from exon 2. The remaining 27 amino acids in each signal peptide are from exon 3. For **A** and B, identities are noted by dots, a dash indicates no residue, and X depicts an unknown amino acid. The color of rat IGF1 amino acids corresponds with the map in Fig 1C.

In rodents and in humans, there are two different IGF1 signal peptides. In both species a common COOH-terminal 27-residue segment is encoded by exon 3, and unique NH_2_-terminal fragments by exon 1 (21 amino acids) or exon 2 (5 amino acids, Fig 1C). In all 25 mammals, a 47–49 residue signal peptide derived from exons 1 and 3 could be identified, and was fairly well conserved, although in all species except mouse (1 difference), there were at least 4 substitutions compared with rat (Fig 3A, left panel, and 3B). The most divergent signal peptides were found in platypus and in the marsupials, in which either 12 or 13 changes were noted from rat, although DNA data for the COOH-terminal part of the wallaby signal peptide was not available in any database (Fig 3). It is unknown how these alterations might affect any aspect of signal peptide function. The NH_2_-terminal part of the signal peptide encoded by exon 2 consists of 5 amino acids in the 18 mammals in which it was detected (Table 4), but only in guinea pig was the predicted sequence identical to the rat. In the other 5 species with an identified exon 2, no open reading frame was found. However, even without an in-frame methionine codon in exon 2 in rabbit, squirrel, opossum, platypus, and Tasmanian devil, the same open reading frame would be maintained in *IGF1*/*Igf1* transcripts containing exon 2 because of the presence of another methionine at the beginning of the common signal peptide encoded in exon 3 (Fig 2B).

**Fig 4:**
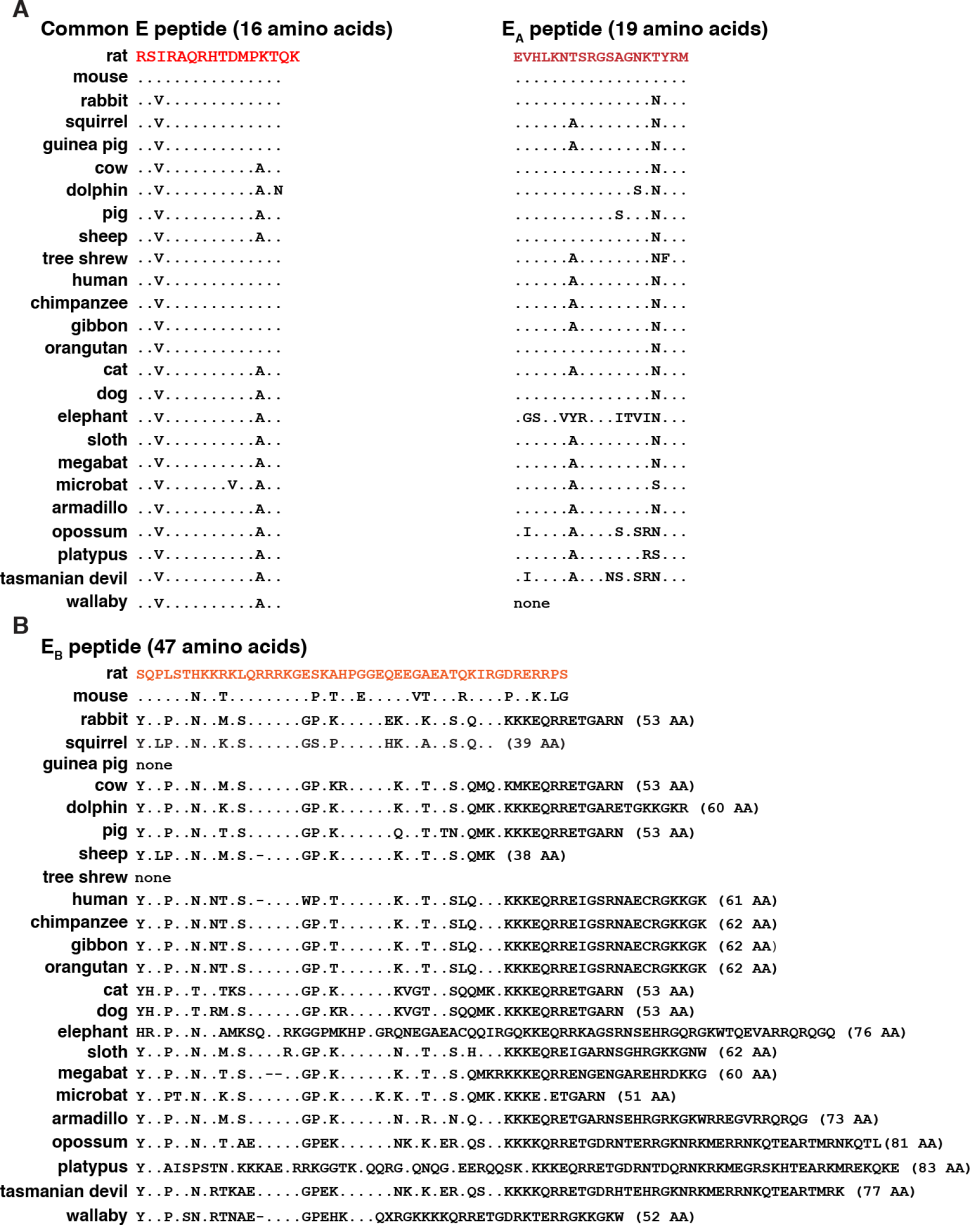
Alignments of mammalian IGF1 precursor peptides. **A**. Amino acid sequences of COOH-terminal common E and E_A_ peptides. **B**. Amino acid sequences of COOH-terminal E_B_ peptides. For **A** and B, identities are indicated by dots and a dash depicts no residue. The number of amino acids in each E_B_ segment is listed in parenthesis. The color of rat IGF1 amino acids corresponds with the map in Fig 1C.

At the COOH-terminal end of the IGF1 protein precursor, the common E region (16 amino acids) and E_A_ peptide (19 residues) were similar to the same parts of rat IGF1, with only 2 to 4 substitutions being observed in 16 species (Fig 4A). However, no E_A_ peptide could be detected in microbat or wallaby, and sequence divergence was greater than 20% in elephant, opossum, and Tasmanian devil (Fig 4A).

**Fig 5:**
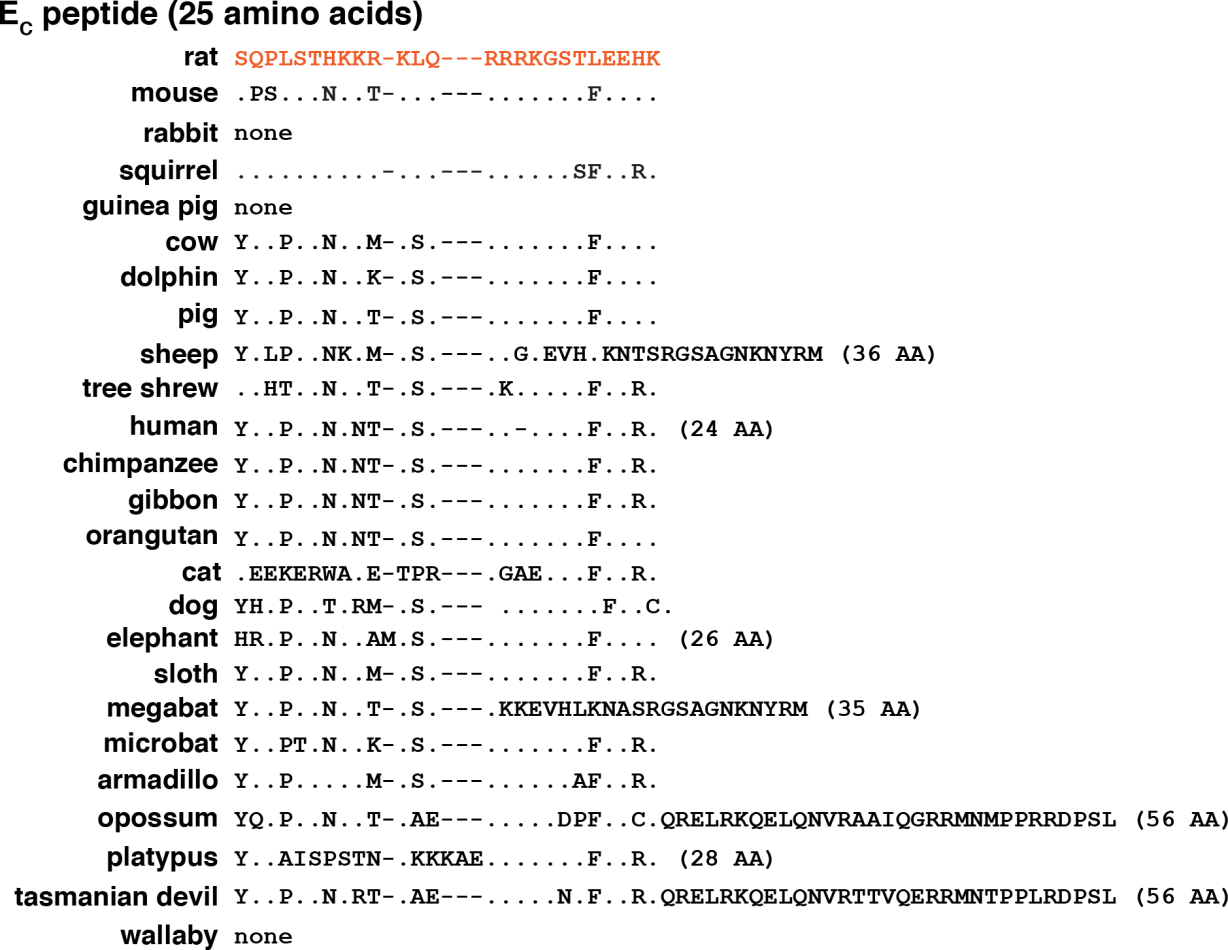
Alignments of mammalian IGF1 E_C_ peptides. Depicted are amino acid sequences of COOH-terminal E_C_ peptides. Identities with rat IGF1 are indicated by dots and a dash depicts no residue. The number of amino acids in each E_C_ segment is listed in parenthesis. The color of rat IGF1 amino acids corresponds with the map in Fig 1C.

The E_B_ segment, which is encoded by exon5, and the E_C_ region, encoded by exons 5 plus 6, were less conserved than other parts of IGF1 precursor proteins among the different mammals evaluated (Figs 4B and 5). The E_B_ peptide ranged from 39 to 83 amino acids in length (Fig 4B), and E_C_ from 25 to 56 residues (Fig 5). In all species studied, both segments are highly enriched in basic amino acids (Figs 4B and 5). Although the reasons for the extensive diversification of these regions compared with other parts of the predicted IGF1 precursor [2, 26] are unknown, it has been postulated that this variability may be secondary to insertion of a transposon from the mammalian interspersed repetitive-b family into the genome at the *IGF1* exon 5 site of a common mammalian ancestor [46]. Of note, there was no E_B_ segment detected in guinea pig or tree shrew, and no E_C_ region in rabbit, guinea pig, or wallaby.

### Insights into *IGF1* gene regulation

Limited studies during the past three decades have identified parts of human *IGF1* and rat *Igf1* gene promoters that are important for their basal activity [17, 47–50], and also have characterized portions of promoter 1 that could mediate actions of the hepatic-enriched transcription factors, HNF-1, HNF-3, and C/EBPα and β on *IGF1* gene transcription in the liver [51–53]. Other DNA elements also have been mapped in human *IGF1* and rat *Igf1* promoter 1 that have been found to serve as response elements for hormones that activate cAMP through the transcription factor, C/EBPδ [54, 55].

**Fig 6:**
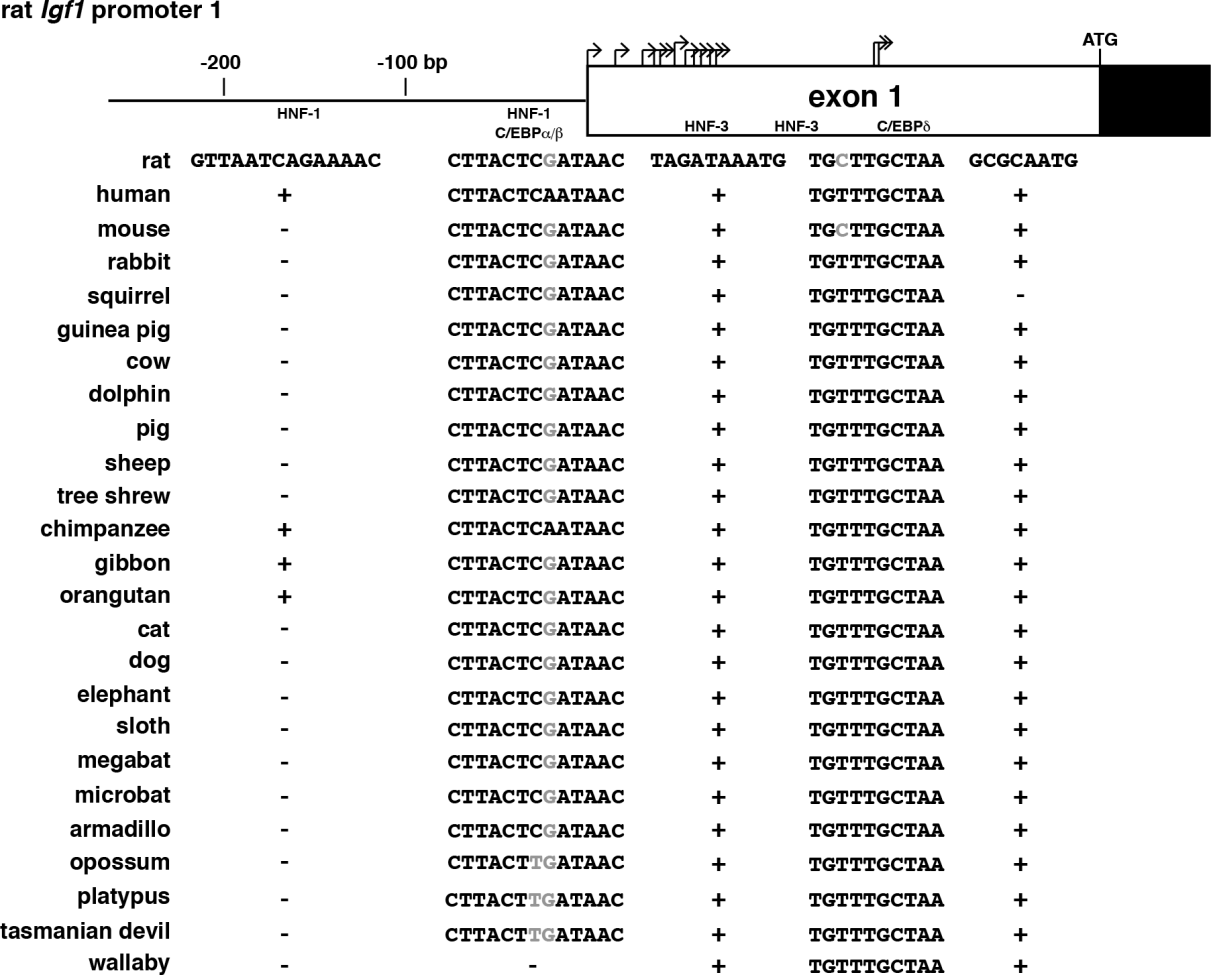
Comparison of *IGF1* promoter 1. **A**. Schematic of rat *Igf1* gene promoter 1 and exon 1. Bent arrows indicate transcription start sites in exon 1, and the location of the ATG codon is labeled. Coding DNA is in black and noncoding in white. The relative frequency of transcription start site usage is depicted by the height of each bent arrow. The locations of binding sites for the transcription factors HNF-1, C/EBPα/β, HNF-3, and C/EBPδ are indicated. The presence of identical sites in different species is indicated by + for each transcription factor site, and the absence is depicted by -; altered nucleotides are shaded in gray.

Presented in Table 3 and depicted in Fig 6 are results of analyses comparing the two rat *Igf1* promoters with their orthologous regions in 24 other mammalian species. In nearly all species, nucleotide conservation was high in the most proximal parts of each promoter, and overall DNA sequence identity ranged from 85% to 92%, depending on the specific genome (Table 3). Moreover, some of the DNA elements described above that have been mapped in proximal human *IGF1* or rat *Igf1* promoter 1 or in noncoding region of exon 1 were highly conserved in the other species. Of particular note are binding sites for C/EBPδ and for HNF-3 located in distal exon 1 and found in nearly all species (Fig 6). This contrasts with the more 5’ HNF-1 site in promoter 1 that was detected only in rat, human, and other primates (Fig 6).

The most important physiological activator of *IGF1/Igf1* is GH [56]. GH stimulates *Igf1* gene transcription in rats via interactions of up to seven inducible Stat5b binding elements that are located throughout the locus, being found in far distal 5’ flanking DNA and in introns, but not near either *Igf1* promoter [37, 38]. These elements have been shown *in vivo* in rat liver to bind Stat5b and several transcriptional co-factors, including p300, RNA polymerase II, and the mediator complex, to undergo reversible histone modifications [37], and at least in cell culture experiments, to physically interact with *Igf1* promoters in a GH-regulated way [57]. These elements thus appear to be *bona fide* transcriptional enhancers [58, 59]. Five of these segments also have been shown to be conserved and to be present in analogous regions in human *IGF1* [39], and in several other non-human primates [14].

**Fig 7:**
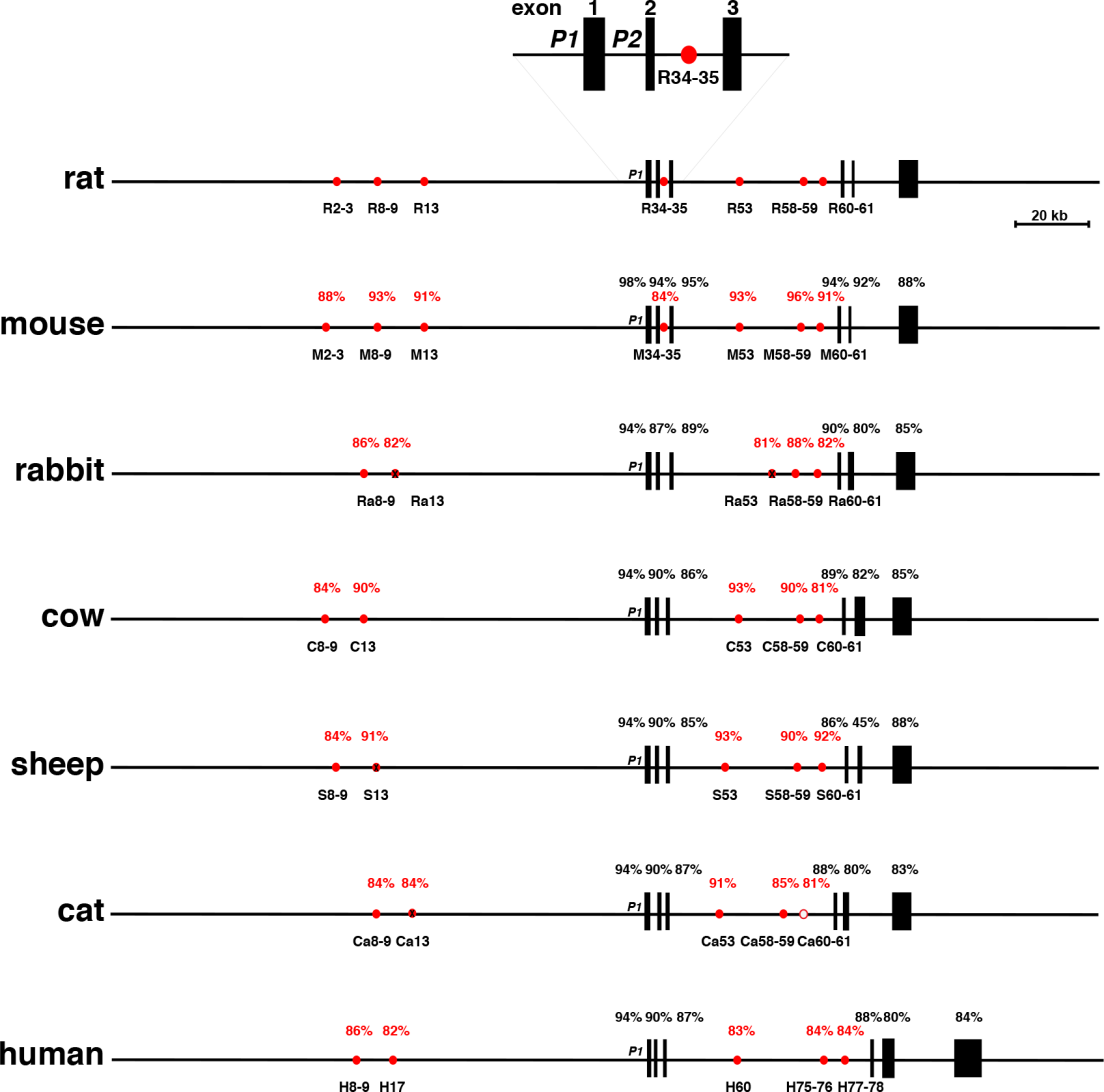
Comparison of mammalian *IGF1/Igf1* genes and loci. Schematics of rat *Igf1*, and selected other *IGF1*/*Igf1* genes and loci are shown. Exons are depicted as boxes, and introns and flanking DNA as horizontal lines. The enlargement above the main map depicts the two rat *Igf1* promoters, P1 and P2, and exons 1–3. Red circles represent locations of sites shown to bind Stat5b in a GH-inducible way in the rat *Igf1* locus, and their homologues in other species. A filled circle indicates the presence of intact Stat5b binding elements, an open circle depicts that one Stat5b site is absent, and an X within a circle represents the absence of all Stat5b binding sequences (see Tables 5 and 6). The percentage of nucleotide identity with different parts of rat *Igf1* is indicated within each gene and locus (black for exons, red for putative Stat5b binding elements). Other abbreviations are as follows: R2-3, R8-9, R13, R34-35, R53-54, R57-59, R60-61-78—nomenclature for rat Stat5b sites (see (37)). A similar nomenclature has been adopted for other species.

**Fig 8:**
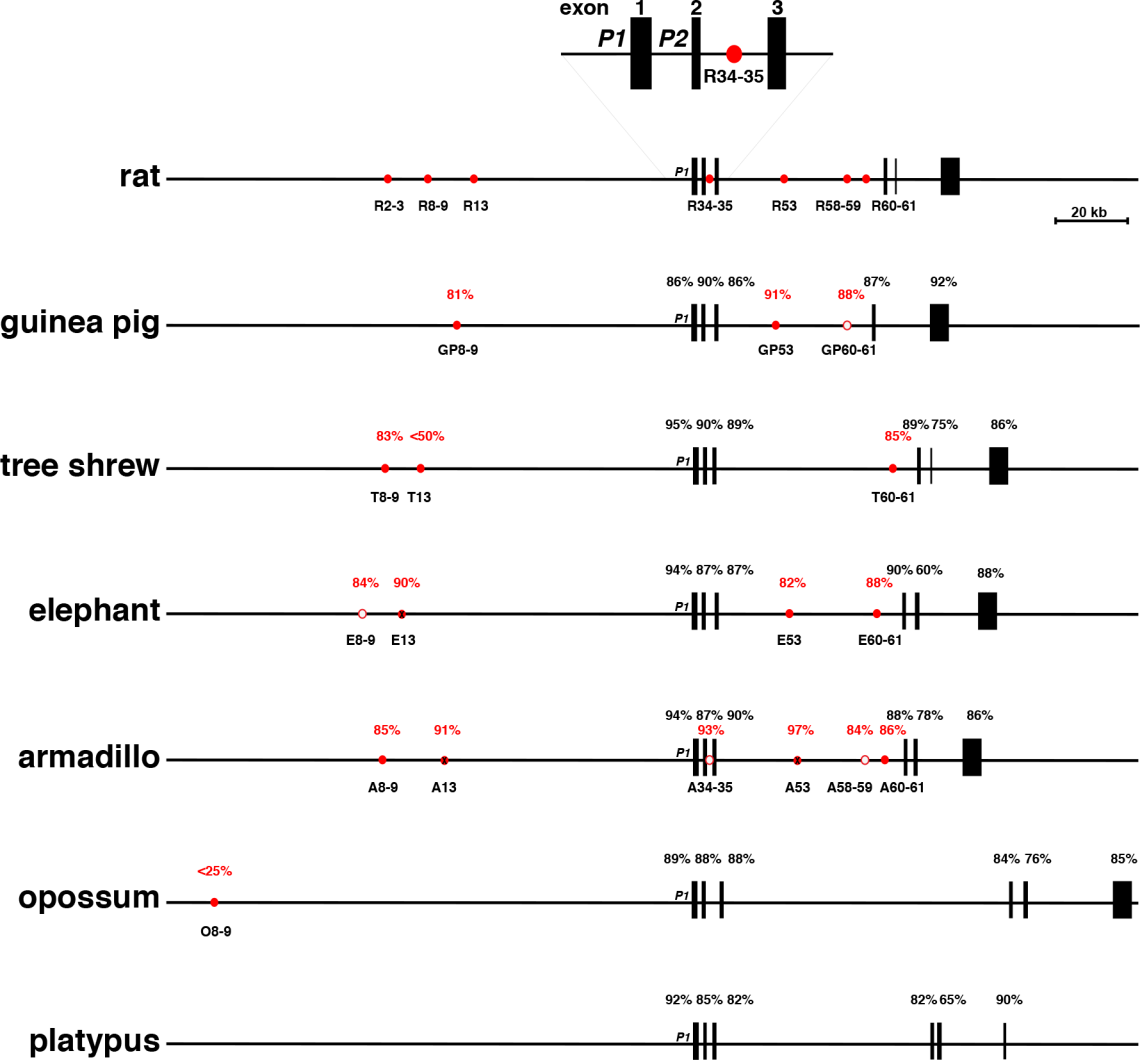
Comparison of additional mammalian *IGF1/Igf1* genes and loci. Maps of rat *Igf1*, and selected other *IGF1*/*Igf1* genes and loci are illustrated. See the legend to Fig 7 for details.

The same seven elements identified near the rat *Igf1* gene could be variably detected in the genomes of other mammals, and tended to be located within respective *IGF1* loci at genome coordinates analogous to those mapped for rat *Igf1* (Tables 5 and 6, Figs 7 and 8). Comparison of the DNA sequences of these elements revealed varying levels of similarity with the corresponding rat regions, ranging from 84% to 96% identity in all seven segments in mouse, including full conservation of the 9-nucleotide pair canonical Stat5b binding sites, and near identity in DNA spacing between paired elements, to low level DNA sequence similarity in just a single segment (homologue of rat [R] 8–9) in opossum and Tasmanian devil, to no elements in platypus (Tables 5 and 6 and Fig 8). Except for platypus, all other mammals had at least one detectable segment with Stat5b sequences, with four mammals exhibiting 1 or 2, six with 3, and twelve encoding 4 or 5 (Tables 5 and 6, Figs 7 and 8). However, no Stat5 site was detected in the equivalent of R13 in eleven species, or in the homologue of R53 in rabbit, orangutan, and armadillo. In addition, in the elephant homologue of R8-9, a single nucleotide modification has occurred within the more 5’ Stat5b binding sequence, changing it from 5’-TTC TTA GAA-3’ to 5’-TTC TTA G**T**A-3’ (Table 5, Fig 7), and presumably rendering it incapable of binding Stat5b [60–62]. A similar inactivating change was found within the 3’ Stat5b 9-base pair homologue of R60-61 in the cat [5’-TTC ACA GA**C**-3’ (Table 5)]. Also, in the armadillo equivalents of R34-35 and R58-59, in the pig homologue of R34-35, and in the guinea pig equivalent of R60-61, the more 3’ Stat5b site was not detected (Table 5). Other single, double or triple nucleotide modifications were found, particularly in cow (R13), in elephant (R53), in fourteen species in R58-59, and in sixteen species in R60-61 (Table 5), but these all are observed in authentic Stat5b binding elements (60–62).

**Table 5:** Comparison of Stat5b binding elements in mammalian *Igf1* loci*.

R2-3** (246 bp)	Rat	Mouse								
% Identity	100	88								
5’ Site	**TTCATGGAA**	**TTCATGGAA**								
Inter-site length (bp)	64	64								
3’ Site	**TTCCTGGAA**	**TTCCTGGAA**								
**R8-9** **(351 bp)**	Rat	Mouse	Rabbit	Squirrel	Guinea Pig	Cow	Dolphin	Pig	Sheep	Tree Shrew
% Identity	100	92.7	85.9	84.8	81.0	84.2	82.9	86.1	84.4	83.4
5’ Site	**TTCTAAGAA**	**TTCTAAGAA**	**TTCTAAGAA**	**TTCTAAGAA**	**TTCTAAGAA**	**TTCTAAGAA**	**TTCTAAGAA**	**TTCTAAGAA**	**TTCTAAGAA**	**TTCTAAGAA**
Inter-site length (bp)	226	223	219	222	205	217	218	218	217	224
3’ Site	**TTCTTAGAA**	**TTCTTAGAA**	**TTCTTAGAA**	**TTCTTAGAA**	**TTCTTAGAA**	**TTCTTAGAA**	**TTCTTAGAA**	**TTCTTAGAA**	**TTCTTAGAA**	**TTCTTAGAA**
**R8-9** **(351 bp)**	Rat	Human	Chimp	Gibbon	Orangutan	Cat	Dog	Elephant	Sloth	Megabat
% Identity	100	86.4	86.4	86.8	86.1	84.2	84.2	83.7	83.0	85.5
5’ Site	**TTCTAAGAA**	**TTCTAAGAA**	**TTCTAAGAA**	**TTCTAAGAA**	**TTCTAAGAA**	**TTCTAAGAA**	**TTCTAAGAA**	**TTCTAAG***T***A**	**TTCTAAGAA**	**TTC***C***AAGAA**
Inter-site length (bp)	226	220	220	220	220	216	217	222	220	207
3’ Site	**TTCTTAGAA**	**TTCTTAGAA**	**TTCTTAGAA**	**TTCTTAGAA**	**TTCTTAGAA**	**TTCTTAGAA**	**TTCTTAGAA**	**TTCTTAGAA**	**TTCTTAGAA**	**TTCTTAGAA**
**R8-9** **(351 bp)**	Rat	Microbat	Armadillo	Opossum	Platypus	Tasmanian Devil	Wallaby			
% Identity	100	83.0	85.0	<25.0	No match	<25.0	80.3			
5’ Site	**TTCTAAGAA**	**TTCTAAGAA**	**TTCTAAGAA**	**TTCTAAGAA**	**-**	**TTCTAAGAA**	**TTCTAAGAA**			
Inter-site length (bp)	226	206	220	224	-	224	221			
3’ Site	**TTCTTAGAA**	**TTCTTAGAA**	**TTCTTAGAA**	**TTCTTAGAA**	**-**	**TTCTTAGAA**	**TTCTTAGAA**			
**R13** **(297 bp)**	Rat	Mouse	Rabbit	Squirrel	Guinea Pig	Cow	Dolphin	Pig	Sheep	Tree Shrew
% Identity	100	90.9	81.5	93.3 (75 bp)	No match	89.9 (69 bp)	86.7 (60 bp)	86.2 (109 bp)	91.3 (69 bp)	<50
5’ Site	**TTCCTTGAA**	**TTCCTTGAA**	**none**	**none**	**-**	**TTC***T***T***A***GAA**	**none**	**none**	**none**	**TTCCTTGAA**
Inter-site length (bp)	-	-	-	-	-	217	-	-	-	-
3’ Site	**none**	**none**	**none**	**none**	**-**	**none**	**none**	**none**	**none**	**none**
**R13** **(297 bp)**	Rat	Human	Chimp	Gibbon	Orangutan	Cat	Dog	Elephant	Sloth	Megabat
% Identity	100	82.4	81.9	84.3 (108 bp)	81.4	84.3 (108 bp)	85.4 (89 bp)	84.9 (99 bp)	No match	88.4 (69 bp)
5’ Site	**TTCCTTGAA**	**TTCCTTGAA**	**TTCCTTGAA**	**TTCCTTGAA**	**TTCCTTGAA**	**none**	**none**	**none**	**-**	**none**
Inter-site length (bp)	-	-	-	-	-	-	-	-	-	-
3’ Site	**none**	**none**	**none**	**none**	**none**	**none**	**none**	**none**	**-**	**none**
**R13** **(297 bp)**	Rat	Microbat	Armadillo	Opossum	Platypus	Tasmanian Devil	Wallaby			
% Identity	100	89.9 (69 bp)	91.3 (46 bp)	No match	No match	No match	No match			
5’ Site	**TTCCTTGAA**	**none**	**none**	**-**	**-**	**-**	**-**			
Inter-site length (bp)	-	-	-	-	-	-	-			
3’ Site	**none**	**none**	**none**	**-**	**-**	**-**	**-**			
**R34-35** **(209 bp)**	Rat	Mouse	Rabbit	Squirrel	Guinea Pig	Cow	Dolphin	Pig	Sheep	Tree Shrew
% Identity	100	83.7	No match	No match	No match	No match	No match	93.1 (29 bp)	No match	No match
5’ Site	**TTCCTGGAA**	**TTCCTGGAA**	**-**	**-**	**-**	**-**	**-**	**TTCCTGGAA**	**-**	**-**
Inter-site length (bp)	60	67	-	-	-	-	-	-	-	-
3’ Site	**TTCTTAGAA**	**TTCTTAGAA**	**-**	**-**	**-**	**-**	**-**	**none**	**-**	**-**
R34-35** (209 bp)	Rat	Microbat	Armadillo							
% Identity	100	No match	92.9 (27 bp)							
5’ Site	**TTCCTGGAA**	**-**	**TTCCTGGAA**							
Inter-site length (bp)	60	-	-							
3’ Site	**TTCTTAGAA**	**-**	**none**							
**R53** **(230 bp)**	Rat	Mouse	Rabbit	Squirrel	Guinea Pig	Cow	Dolphin	Pig	Sheep	Tree Shrew
% Identity	100	93.3	81.2	95.2 (41 bp)	90.6 (53 bp)	92.9 (28 bp)	93.3 (30 bp)	No match	92.9 (28 bp)	No match
5’ Site	**TTCAGGGAA**	**TTCAGGGAA**	**none**	**TTCAGGGAA**	**TTCAGGGAA**	**TTCAGGGAA**	**TTCAGGGAA**	**-**	**TTCAGGGAA**	**-**
Inter-site length (bp)	-	-	-	-	-	217	-	-	-	-
3’ Site	**none**	**none**	**none**	**none**	**none**	**TTCTTAGAA**	**none**	**-**	**none**	**-**
**R53** **(230 bp)**	Rat	Human	Chimp	Gibbon	Orangutan	Cat	Dog	Elephant	Sloth	Megabat
% Identity	100	83.3	83.3	90.8 (65 bp)	83.3	91.3 (46 bp)	85.5 (69 bp)	81.9 (105 bp)	No match	86.4 (59 bp)
5’ Site	**TTCAGGGAA**	**TTCAGGGAA**	**TTCAGGGAA**	**TTCAGGGAA**	**none**	**TTCAGGGAA**	**TTCAGGGAA**	**TTCAG***A***GAA**	**-**	**TTCAGGGAA**
Inter-site length (bp)	-	-	-	-	-	-	-	-	-	-
3’ Site	**none**	**none**	**none**	**none**	**none**	**none**	**none**	**none**	**-**	**none**
**R53** **(230 bp)**	Rat	Microbat	Armadillo	Opossum	Platypus	Tasmanian Devil	Wallaby			
% Identity	100	No match	96.8 (31 bp)	No match	No match	No match	No match			
5’ Site	**TTCAGGGAA**	**-**	**none**	**-**	**-**	**-**	**-**			
Inter-site length (bp)	-	-	-	-	-	-	-			
3’ Site	**none**	**-**	**none**	**-**	**-**	**-**	**-**			
**R58-59** **(271 bp)**	Rat	Mouse	Rabbit	Squirrel	Guinea Pig	Cow	Dolphin	Pig	Sheep	Tree Shrew
% Identity	100	96.1	88.1	92.4 (66 bp)	No match	89.8 (98 bp)	88.0 (100 bp)	88.7 (97 bp)	89.8 (98 bp)	No match
5’ Site	**TTCTCAGAA**	**TTCTCAGAA**	**TTCTCAGAA**	**TTCTCAGAA**	**-**	**TTCTCAGAA**	**TTCTCAGAA**	**TTCTCAGAA**	**TTCTCAGAA**	**-**
Inter-site length (bp)	6	6	6	6	-	6	6	6	6	-
3’ Site	**TTCGCAGAA**	**TTCGCAGAA**	**TTC***A***CAGAA**	**TTC***A***CAGAA**	**-**	**TTC***A***CAGAA**	**TTC***A***CAGAA**	**TTC***A***CAGAA**	**TTC***A***CAGAA**	**-**
**R58-59** **(271 bp)**	Rat	Human	Chimp	Gibbon	Orangutan	Cat	Dog	Elephant	Sloth	Megabat
% Identity	100	84.3	83.8	83.2	84.9	85.1 (94 bp)	85.5 (83 bp)	No match	No match	84.9 (73 bp)
5’ Site	**TTCTCAGAA**	**TTCTCAGAA**	**TTCTCAGAA**	**TTCTCAGAA**	**TTCTCAGAA**	**TTCTCAGAA**	**TTCTCAGAA**	**-**	**-**	**TTCTC***G***GAA**
Inter-site length (bp)	6	6	6	6	6	6	6	-	-	6
3’ Site	**TTCGCAGAA**	**TTC***A***CAGAA**	**TTC***A***CAGAA**	**TTC***A***CAGAA**	**TTC***A***CAGAA**	**TTC***ATG***GAA**	**TTC***ATG***GAA**	**-**	**-**	**TTC***AT***AGAA**
**R58-59** **(271 bp)**	Rat	Microbat	Armadillo	Opossum	Platypus	Tasmanian Devil	Wallaby			
% Identity	100	88.4 (95 bp)	83.6 (61 bp)	No match	No match	No match	No match			
5’ Site	**TTCTCAGAA**	**TTCTCAGAA**	**TTCTCAGAA**	**-**	**-**	**-**	**-**			
Inter-site length (bp)	6	6	-	-	-	-	-			
3’ Site	**TTCGCAGAA**	**TTC*A*CAGAA**	**none**	**-**	**-**	**-**	**-**			
**R60-61** **(329 bp)**	Rat	Mouse	Rabbit	Squirrel	Guinea Pig	Cow	Dolphin	Pig	Sheep	Tree Shrew
% Identity	100	91.3	81.8	84.6	87.7 (122 bp)	81.2	92.5 (40 bp)	82.0	92.3 (39 bp)	84.7
5’ Site	**TTCCTAGAA**	**TTCCTAGAA**	**TTCCTAGAA**	**TTCCTAGAA**	**TTCCTAGAA**	**TTCCTAGAA**	**TTCCTAGAA**	**TTCCTAGAA**	**TTCCTAGAA**	**TTCCTAGAA**
Inter-site length (bp)	127	126	128	127	-	126	126	125	124	127
3’ Site	**TTCACAGAA**	**TTCACAGAA**	**TTCA***T***AGAA**	**TTCACAGAA**	**none**	**TTCA***T***AGAA**	**TTCA***T***AGAA**	**TTCA***T***AGAA**	**TTCA***T***AGAA**	**TTCA***T***AGAA**
**R60-61** **(329 bp)**	Rat	Human	Chimp	Gibbon	Orangutan	Cat	Dog	Elephant	Sloth	Megabat
% Identity	100	84.3	83.8	84.8	84.8	81.4	82.5 (120 bp)	90.1 (98 bp)	86.2 (123 bp)	83.6 (140 bp)
5’ Site	**TTCCTAGAA**	**TTCCTAGAA**	**TTCCTAGAA**	**TTCCTAGAA**	**TTCCTAGAA**	**TTCCTAGAA**	**TTCCTAGAA**	**TTCCTAGAA**	**TTCCTAGAA**	**TTCCTAGAA**
Inter-site length (bp)	127	128	128	128	128	126	126	126	370	126
3’ Site	**TTCACAGAA**	**TTCA***T***AGAA**	**TTCA***T***AGAA**	**TTCA***T***AGAA**	**TTCA***T***AGAA**	**TTCACAGA***C*	**TTCA***T***AGAA**	**TTCA***T***AGAA**	**TTCA***T***AGAA**	**TTCA***T***AGAA**
**R60-61** **(329 bp)**	Rat	Microbat	Armadillo	Opossum	Platypus	Tasmanian Devil	Wallaby			
% Identity	100	84.5 (116 bp)	86.2 (61 bp)	No match	No match	No match	No match			
5’ Site	**TTCCTAGAA**	**TTCCTAGAA**	**TTCCTAGAA**	**-**	**-**	**-**	**-**			
Inter-site length (bp)	127	126	127	-	-	-	-			
3’ Site	**TTCACAGAA**	**TTCA***T***AGAA**	**TTCA***T***AGAA**	**-**	**-**	**-**	**-**			

*Italic text indicates mis-matched nucleotide

**No other matches identified

**Table 6:** Comparison of Stat5b elements in *IGF1/Igf1* loci.

Species	R2-3	R8-9	R13	R34-35	R53	R58-59	R60-61	Total sites
Rat	2 sites	2 sites	1 site	2 sites	1 site	2 sites	2 sites	12
Mouse	2	2	1	2	1	2	2	12
Rabbit	0	2	0	0	0	2	2	6
Squirrel	0	2	0	0	1	2	2	7
Guinea pig	0	2	0	0	1	0	1	4
Cow	0	2	1	0	1	2	2	8
Dolphin	0	2	0	0	1	2	2	7
Pig	0	2	0	1	0	2	2	7
Sheep	0	2	0	0	1	2	2	7
Tree shrew	0	2	1	0	0	0	2	5
Human	0	2	1	0	1	2	2	8
Chimpanzee	0	2	1	0	1	2	2	8
Gibbon	0	2	1	0	1	2	2	8
Orangutan	0	2	1	0	0	2	2	7
Cat	0	2	0	0	1	2	2	7
Dog	0	2	0	0	1	2	1	6
Elephant	0	1	0	0	1	0	2	4
Sloth	0	2	0	0	0	0	2	4
Megabat	0	2	0	0	1	2	2	7
Microbat	0	2	0	0	0	2	2	6
Armadillo	0	2	1	0	0	1	2	6
Opossum	0	2	0	0	0	0	0	2
Platypus	0	0	0	0	0	0	0	0
Tas. devil	0	2	0	0	0	0	0	2
Wallaby	0	2	0	0	0	0	0	2

To date the mechanisms responsible for the patterns of alternative splicing of *IGF1*/*Igf1* transcripts in different cell types are unknown. Examination of human *IGF1* mRNAs in GTEx has revealed marked variation in steady-state levels of transcripts containing just exon 5 (40 to 65%), exon 6 (40 to 55%), or both exons 5 and 6 (2 to 10% of all mRNAs) in the 37 different organs and tissues in the database. Variation is also observed in the fraction of mRNAs containing these different exon combinations in mouse and rat tissues in the SRA NCBI.

### The sloth *IGF1* locus contains a processed pseudogene

**Fig 9:**
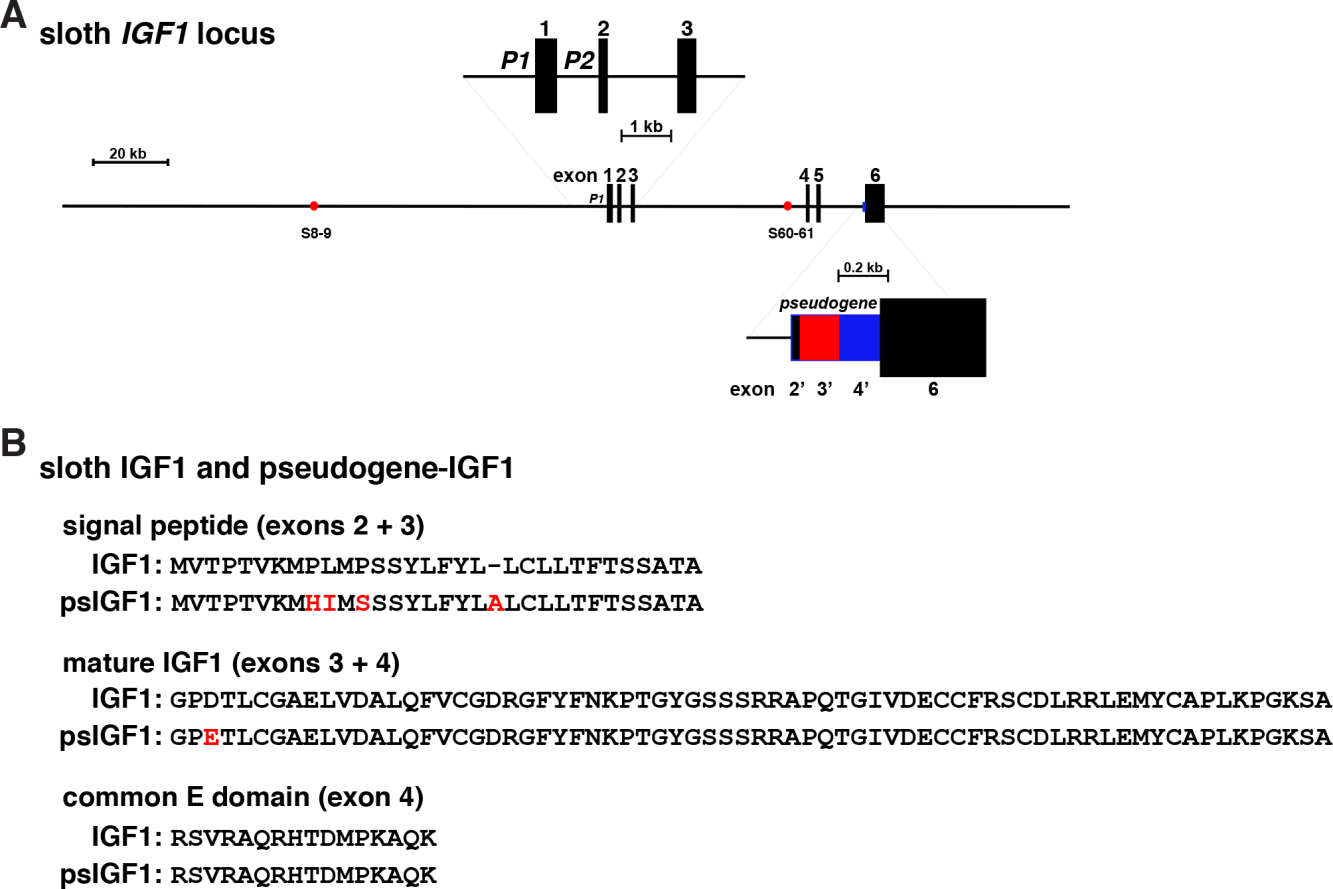
The sloth *IGF1* locus encodes a processed pseudogene. **A**. Map of the sloth *IGF1* gene and locus. Exons are depicted as boxes, and introns and flanking DNA as horizontal lines. The enlargement above the main map illustrates the two *Igf1* promoters, P1 and P2, and exons 1–3. Red circles represent locations of homologues of sites shown to bind Stat5b in a GH-inducible way in the rat *Igf1* locus. The enlargement below the main map depicts the location and structure of the putative *IGF1* pseudogene, which consists of DNA segments that are nearly identical to sloth IGF1 exons 2, 3, and 4 (color coded in black, red, and blue, and marked as exons 2’, 3’, and 4’, respectively). **B**. Comparison of amino acid sequences of sloth IGF1 with sloth pseudogene-IGF1 in single letter code. Differences are depicted in red; a dash indicates no residue.

Initial screening of the sloth genome with rat *Igf1* revealed two DNA sequences with similar levels of identity with rat exons 3 and 4 (87% and 86%, respectively). Two of these segments mapped ~50 kb apart in the sloth genome (Table 1), and the other two were adjacent to one another, and were located immediately 5’ to the beginning of sloth *IGF1* exon 6 (Fig 9A). Further analysis revealed that contiguous with and 5’ to the alternative exon 3 were 52 base pairs that were identical with the 3’ part of sloth *IGF1* exon 2, and included the 5 codon open reading frame. Collectively, this 391-nucleotide pair genomic segment was over 98% identical to sloth *IGF1* exons 2–4. Conceptual translation of an mRNA predicted from these DNA sequences revealed marked similarity with the sloth IGF1 protein precursor, with only four mismatches in the common signal peptide, one in mature 70-residue IGF1, and none in the common E peptide, for an overall amino acid identity of nearly 96% with the authentic sloth IGF1 precursor (Fig 9B). It seems likely that this represents a processed mRNA that was retro-transposed as a DNA copy back into the sloth IGF1 locus [63]. Alternatively, it is possible that these results reflect some inaccuracies within the incomplete sloth genome. Since this putative pseudogene maps to the 5’ end of sloth *IGF1* exon 6, it is possible that the postulated mRNA from which this DNA segment derives also included exon 6, although against this argument it should be noted that there is no duplication of authentic sloth *IGF1* exon 6 in the genome. *IGF1* pseudogenes have not been recognized in mammals to date, yet there is a precedent with the gene for the related peptide insulin in mice and rats. The *Ins1* gene appears to have been derived from *Ins2* by an analogous mechanism, although in this case, one of two introns was retained in *Ins1* and the new and still-functional gene was not inserted within the Ins2 locus [64].

## Discussion

Public biological databases represent rich sources of information about genes from various organisms, and in depth analysis of these data can be the impetus for the development of new hypotheses on evolutionary aspects of gene structure, function, or regulation. This report focuses on the molecular genetics of *IGF1*, as seen through the lens of 25 mammalian species. These genomes were chosen because they represent 15 different orders and cover ~180 million years of evolutionary diversification, although a different cohort of the approximately 100 different mammalian species whose DNA sequences are available might have yielded an analogous data set. It is generally thought that IGF1, a 70-amino acid single-chain secreted protein, plays a central role in regulating somatic growth during childhood in humans and in juveniles of other mammals, and functions as both a mediator of the actions of GH [5–9], and as a readout for the environmental inputs that affect overall health [65]. IGF1 also is involved in tissue repair and in metabolic regulation throughout life [10–13]. In humans, rats, and mice, the protein precursor of mature IGF1 is derived from the translation of multiple classes of mRNAs that result from transcription from two distinct promoters using several different initiation sites, and alternative RNA splicing [2, 19] (see Fig 1B). The genomic analyses presented here suggest that similar mechanisms are active in at least 20 other mammalian species from 11 additional orders. The genomes of these species all encode single-copy *IGF1* genes that share structural features with human *IGF1* and rat *Igf1*, namely two promoters, and six exons subdivided by five introns, including a central large intron of ~47 - ~80 kb separating exons 3 and 4 (Figs 7 and 8, Tables 1–3). Nearly all of these *IGF1* genes also appear capable of being transcribed and processed into many classes of *IGF/Igf1* mRNAs similar to those found in humans and rats [15, 16, 19, 22–25], of being translated into the same types of IGF1 precursor proteins, and of being processed into a highly similar mature IGF1 peptide (Fig 2, Table 4). Regarding the other 2 species with apparently different *IGF1* genes, in guinea pig, a homologue of exon 5 was not identified, and in wallaby, the DNA quality of the *IGF1* locus was poor in all databases that were searched (Tables 1–3). Thus, it is likely that in the majority of mammals, *IGF1* is a 2-promoter, 6-exon, and 5-intron gene.

There also is fairly extensive DNA sequence identity in the proximal parts of the two *IGF1* promoters among most of the 25 mammalian species evaluated here. This includes conservation of transcription factor binding sites for HNF-1, C/EBPα/β, HNF-3, and C/EBPδ in promoter 1 in most species (Fig 6). These data suggest that common regulatory mechanisms may control some aspects of *IGF1* gene expression in the majority of mammals.

A more surprising result of analysis of many mammalian *IGF1* genes is the apparent divergence of putative GH-regulated Stat5b binding enhancer elements in *IGF1* loci (Figs 7 and 8, Tables 5 and 6). Since GH plays a critical role in the molecular physiology of IGF1 in several mammalian species [20, 31, 36, 37], and since in previous studies, conserved GH-inducible Stat5b-binding enhancer elements were identified in analogous locations in the *IGF1*/*Igf1* loci of humans, several non-human primates, rats, and mice [14, 37, 39, 66], it was assumed that similar DNA segments would be shared among other mammals. With results from additional species, it now appears that the number of recognizable putative GH-responsive Stat5b-binding elements varies considerably (Tables 5 and 6, Figs 7, 8 and 9). Since no potential Stat5b-binding domains were detected in the platypus *IGF1* locus, and only a single element with a pair of Stat5b sites was found in opossum, Tasmanian devil, and wallaby (Tables 5 and 6, Fig 8), these results suggest that during mammalian speciation, particularly of marsupials and monotremes, either other mechanisms have arisen to govern GH-activated *IGF1* gene transcription (e.g., Stat5b-binding sequences located elsewhere in the loci), or alternatively, GH does not stimulate *IGF1* gene expression in these species. Since it is now possible to construct chimeric cell lines containing *IGF1* loci from different species and test for GH-regulated transcription [39, 57], these different hypotheses may be examined directly.

Ensembl, the SRA NCBI, and other publically available genomic databases contain a wealth of information about different genes from a wide range of animal species, yet much of these data have not been analyzed fully or even characterized yet. For most of the *IGF1* genes and loci examined here, the information found within Ensembl was either incompletely or incorrectly annotated, possibly because the analyses were limited in scope or were not reviewed by anyone with expertise in the molecular biology of this gene. Similarly, the data in the SRA have not been evaluated in any detail, and all human RNA samples in GTEx are derived from post-mortem tissues and organs. It seems likely that similar situations as seen with *IGF1*/*Igf1* exist for other genes, raising the possibility that there are many opportunities to gain new insights into gene conservation or variation during mammalian and vertebrate evolution. For example, there may be other species besides the sloth in which an *IGF1* RNA copy was retro-transposed as DNA back into the *IGF1* locus (Fig 9) or even elsewhere in the genome.

As described in detail in humans [67], it is likely that most other mammalian genomes contain several million DNA sequence polymorphisms, and that at least some of these modifications have the potential to alter gene expression based on their locations within enhancers, promoters, or other regulatory components [68]. For example, single nucleotide polymorphisms (SNPs) have been identified in human *IGF1* promoter 1 [69], in at least three Stat5b binding elements (39), and in other parts of the locus (see the database on human variation in GTEx), although to date none of these changes have been studied to determine if they alter *IGF1* gene expression or regulation. Similar data on genomic variability have not been mapped for other mammals, primarily because of the small number of genomes that have been sequenced. It seems likely that analogous differences will be found, including SNPs, copy-number variations, and DNA insertion-deletions that may play roles in population fitness or other adaptations to changing environments. It also seems plausible that certain polymorphic variants may be present in several closely related mammalian species, giving rise to the hypothesis that they have contributed to organismal fitness of common ancestors.

The complicated role of IGF1 in both normal physiology and in disease is potentially reflected in its complex structure and patterns of expression. The fairly high conservation of the *IGF1* gene and protein among the species studied here supports the idea that analogous transcriptional and other regulatory pathways have been present since the onset of mammalian speciation [70, 71], ideas that now can be tested in experimental systems with the expectation that they will lead to new insights into the comparative biology of IGF1 and GH signaling or action.
